# Development of a pro-arrhythmic ex vivo intact human and porcine model: cardiac electrophysiological changes associated with cellular uncoupling

**DOI:** 10.1007/s00424-020-02446-6

**Published:** 2020-09-01

**Authors:** Joseph Brook, Min-young Kim, Simos Koutsoftidis, David Pitcher, Danya Agha-Jaffar, Annam Sufi, Catherine Jenkins, Konstantinos Tzortzis, Suofeiya Ma, Richard J. Jabbour, Charles Houston, Balvinder S. Handa, Xinyang Li, Ji-Jian Chow, Anand Jothidasan, Poppy Bristow, Justin Perkins, Sian Harding, Anil A Bharath, Fu Siong Ng, Nicholas S Peters, Chris D Cantwell, Rasheda A Chowdhury

**Affiliations:** 1 Faculty of Medicine, National Heart and Lung Institute, Imperial College London, Hammersmith Campus, Du Cane Road, London, W12 0NN UK; 2 Faculty of Engineering, Imperial College London, South Kensington Campus, Exhibition Road, London, SW7 2AZ UK; 3 Harefield Hospital, Hill End Road, Harefield, UB9 6JH UK; 4 Royal Veterinary College, University of London, Hawkshead Lane, Hertfordshire, AL97TA UK

**Keywords:** Langendorff, Ex vivo model, Isolated heart, Contact electrogram, Gap junction uncoupling, Carbenoxolone

## Abstract

We describe a human and large animal Langendorff experimental apparatus for live electrophysiological studies and measure the electrophysiological changes due to gap junction uncoupling in human and porcine hearts. The resultant ex vivo intact human and porcine model can bridge the translational gap between smaller simple laboratory models and clinical research. In particular, electrophysiological models would benefit from the greater myocardial mass of a large heart due to its effects on far-field signal, electrode contact issues and motion artefacts, consequently more closely mimicking the clinical setting. Porcine (*n* = 9) and human (*n* = 4) donor hearts were perfused on a custom-designed Langendorff apparatus. Epicardial electrograms were collected at 16 sites across the left atrium and left ventricle. A total of 1 mM of carbenoxolone was administered at 5 ml/min to induce cellular uncoupling, and then recordings were repeated at the same sites. Changes in electrogram characteristics were analysed. We demonstrate the viability of a controlled ex vivo model of intact porcine and human hearts for electrophysiology with pharmacological modulation. Carbenoxolone reduces cellular coupling and changes contact electrogram features. The time from stimulus artefact to (-dV/dt)_max_ increased between baseline and carbenoxolone (47.9 ± 4.1–67.2 ± 2.7 ms) indicating conduction slowing. The features with the largest percentage change between baseline and carbenoxolone were fractionation + 185.3%, endpoint amplitude − 106.9%, S-endpoint gradient + 54.9%, S point − 39.4%, RS ratio + 38.6% and (-dV/dt)_max_ − 20.9%. The physiological relevance of this methodological tool is that it provides a model to further investigate pharmacologically induced pro-arrhythmic substrates.

## Background

### Ex vivo models

A major challenge for translational research in the cardiovascular field is to effectively and efficiently translate research from bench to bedside. In vitro and small animal disease models in particular are limited in their recapitulation of the human phenotype, with only a third of small animal research studies resulting in positive translation to human randomised trials [13]. Cell and small animal models are still an absolute necessity in disease characterisation and safety assessment before scaling up. This is due to their ability to control confounding factors and their relative simplicity. However, the limitations of these models must be recognised when extrapolating findings from such studies to human hearts [12]. Cell models lack the myocardial mass to represent the effects found in intact preparations, whilst smaller animal models are anatomically and physiologically less comparable with the clinic than large animals. Clinical research on the other hand has the disadvantage that ethical and practical considerations limit the procedures and techniques which can be performed. Therefore, full characterisation and validation of cellular factors and mechanistic insight cannot be obtained.

Explanted intact human and porcine hearts provide the ideal middle ground to bridge this translational gap in arrhythmia research between simpler laboratory models and patients. Despite the advantages of such models, the ethical and practical challenges of procuring these samples and sustaining them in the laboratory have resulted in limited success of implementing the model. The procedure for isolating and perfusing an intact animal heart ex vivo has remained largely unchanged since it was developed for the frog heart in 1895 [11]. This is due to its efficacy at keeping the preparation viable for several hours [15], which is sufficient for most data acquisition protocols [21]. However, it is predominantly used in rodent animal models only due to the complexities involved in developing large animal and human Langendorff systems, as well as challenges in the acquisition of these organs. Although there have been previous attempts to carry out studies in intact ex vivo human hearts, these have been achieved in only a handful of centres due to the aforementioned challenges [7, 8, 16]. To date, no work has been published on the utility of this model for assessing the electrophysiology changes under pharmacological intervention. This increases the potential use of this model to mimic the pathological substrate.

### The contact electrogram

The contact electrogram (EGM) is the signature of the interaction of electrical activation and architecture of the local myocardium and is therefore the main data source in the clinical cardiac catheter laboratory. During interventional procedures to treat arrhythmias, electrodes between 2 and 4 mm in size are held in direct contact with cardiac tissue and the potential difference relative to a distant electrode is recorded [17]. Healthy myocardium gives rise to an electrogram with relatively simple morphology, but intra-cellular uncoupling or ion channel abnormalities may produce a signal with more complex, or fractionated, morphology [18]. The EGM is categorised clinically by binary classifications, such as *simple*, *complex* or *early*, *late*. This form of classification gives little insight into the content of the signal itself and means much of the information within the EGM is left unused [14]. This is because defining the relationship between the characteristics of the myocardial structure, its electrophysiology and the EGM remains elusive. However, the EGMs produced from more reductionist models which allow for controlled characterisation are very different from those found in patients, since the reduced cardiac myocardial mass lacks far-field signals, motion artefacts and issues with proper contact. To investigate the EGMs in a manner which is translatable to the clinic, we need to overcome these in a controllable model which is as close to in vivo as possible.

### Clinical motivation

Atrial fibrillation (AF) is the most common form of cardiac arrhythmia, affecting around 1.3 million people in the UK and is associated with an increased risk of stroke, heart failure and death [9]. Treatment of AF often involves catheter ablation, which uses localised freezing or radiofrequency energy to electrically isolate or destroy problematic regions of myocardium and prevent the initiation and perpetuation of re-entrant tachycardias or AF. Catheter ablation is the most effective intervention strategy used for treating persistent AF. However, despite attempts to improve the success rates through clinical practice, patients found to be free from the disease after 12–30 months following treatment can range between only 20 and 60% [17].

Acute induction of the physiological consequences of ion channel blockade and cellular uncoupling can be recapitulated by pharmacological interventions. Carbenoxolone (CBX) is one such agent that leads to gap junction uncoupling and conduction discontinuity, therefore modelling pro-arrhythmic properties. The porcine and human Langendorff can be used to create a model comparing baseline with pro-arrhythmic measurements in a controllable laboratory environment. This in turn has the potential to provide a greater understanding and improved interpretation of measurements of the electrophysiological structure and function needed to improve treatment outcomes. For mechanistic insight at a cellular level, we investigated these effects in both the left ventricle and left atrium. In this study, we aimed to demonstrate the utility of the intact ex vivo porcine and human heart as a model for high fidelity, controlled electrophysiological studies with acute pharmacological modulation.

## Methods

### Heart acquisition

Human hearts were acquired once an explanted donor heart within the UK was deemed unsuitable for transplant and when all other avenues of clinical usage had been exhausted (*n* = 4). Information on the cause of death, reason for rejection and ischaemic time was subsequently made available, along with any further clinical data or necessary details (Table 1). The samples were fully anonymised, and researchers had no access to identifiable data. Hearts were transported to the laboratory in cardioplegia on ice by emergency medical courier. Transport time was between 1 and 6 h, depending on location, which is within the window of viability for a heart in cardioplegia [1].

**Table 1:** The age, sex, reason for rejection and cause of death for all of the human donor hearts (*n* = 4)

Heart	Donor age	Donor sex	Reason for rejection	Cause of death
6	32	Female	History of meningitis	Haemorrhage
9	36	Male	High lactate	Drowning
11	51	Male	Poor function	Intracranial bleed
13	20	Male	Malignancy found in pancreas	Traffic accident (unrestrained passenger)

Porcine hearts (*n* = 9) were explanted from healthy large white female pigs, weighing 70–80 kg and 4–5 months old. Briefly, pigs were premedicated with intramuscular administration of Ketamine (20 mg/kg) and Midazolam (0.5 mg/kg), followed by intravenous administration of Propofol (2–4 mg/kg). Anaesthesia was maintained using inhaled Sevoflurane and intravenous constant rate infusion (CRI) of Fentanyl (3 μg/kg/h), followed by removal of the heart using a human donor retrieval protocol [23]. Porcine hearts were also transported in cardioplegia on ice to the laboratory within 1 h.

### Langendorff apparatus

**Fig. 1:**
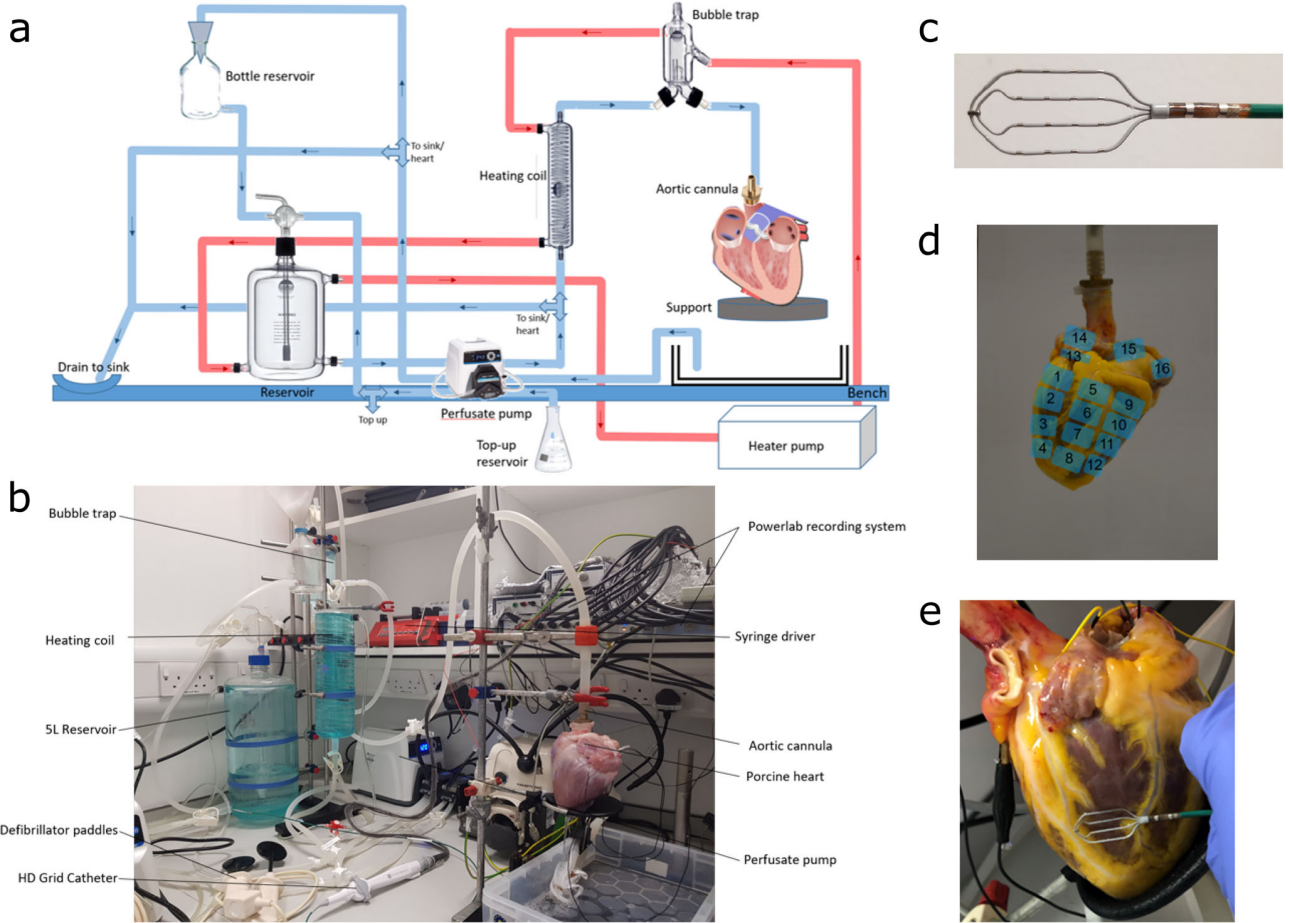
**a** Diagram of the Langendorff apparatus. Blue: tubing for the physiological solution. Red: tubing for the surrounding heating solution. **b** The Langendorff apparatus, pump and aortic cannula feeding into a porcine heart. **c** 4 × 4 HD grid catheter by Abbott Medical, used to record the electrograms from the left ventricle and atrium. **d** The locations on the epicardial surface from where the HD grid catheter was placed to perform the pacing protocol. Positions 1–12 are on the epicardial surface of the left ventricle and positions 13–16 are on the epicardial surface of the left atrium. **e** A photograph of a human heart taken during the pacing protocol at BL to record the location of the catheter when the EGMs were gathered. In this photograph, the catheter is at position 3

A custom-build Langendorff apparatus was constructed to provide the necessary environment to keep the intact human and porcine hearts alive outside the body for over 3 h, which is sufficient for the electrophysiological data acquisition protocols used in this study (Fig. 1a and b). The apparatus consisted of a 5-l solution reservoir (custom supply from Radnoti Ltd.), an oxygen supply to oxygenate the physiological solution to maximal saturation, a heating coil to ensure the solution remains at an optimal temperature of 37 ± 0.5 °C (custom supply from Radnoti Ltd.), a bubble trap to remove air pockets from the system (custom supply from Radnoti Ltd.) and a high flow peristaltic pump to circulate solution around the system at a constant rate (Cole Parmer, UK). The physiological solution was oxygenated Tyrode’s solution, used to flush out the cardioplegia, warm the heart back to optimum temperature levels and continuously provide the heart with the glucose and ions necessary to recommence and then maintain electrical activity and contraction [2].The solution chambers were connected to an aortic cannula, where the normothermic and oxygenated physiological solution was perfused into the coronary arteries in a retrograde manner, thereby maintaining the metabolic, electrical and contractile activity of the heart. The perfusate exited the coronary circulation into the right atrium and was subsequently expelled from the heart. Following the initial period of cardioplegia washout and warming up, the heart was cardioverted if needed using a defibrillator at 10–50 mV. The heart was paced from the basal region of the left ventricle at 10% above the threshold of activation (usually 2 mV) using a clinical stimulator (Micropace EP Inc., USA) and monitored for 15–30 min to ensure full stabilisation and reduction in ST segment elevation prior to recording.

### Monitoring and recording

Following the warm reperfusion and stabilisation time, electrogram recordings were carried out under steady pacing at 2-5A of cycle lengths 300, 400, 500, 660, 750, 1000 and 1500 ms. Clinical HD grid mapping catheters (Abbot Medical) (Fig. 1c), connected to an electrophysiological recording system (PowerLab, AD Instruments) with the Labchart software (AD Instruments), were used to record the electrophysiological activity using a bandwidth of 0.3–500 Hz and a 1-KHz sampling rate from multiple sites across the left ventricular and left atrial epicardium (Fig. 1d). Single-lead electrocardiogram (ECG) in the left ventricle, pressure and temperature were also monitored throughout to assess and enable adjustments to be made as necessary to maintain the stability of the preparation.

Recordings were taken sequentially over 16 areas of the epicardial surface of the left ventricle and the left atrium. A high-resolution photograph is taken at each electrogram site to record the specific position of the catheter’s electrode grid on the epicardial surface (Fig. 1d and e). The positioning of the catheter was superimposed on a 3D reconstruction of the heart after the experiment using photogrammetry. After baseline (BL) recordings at all sites, 50 ml of 1 mM CBX was administered through the aortic cannular, using a syringe driver at a rate of 5 ml/min, to induce gap junction blockade. This resulted in a final circulating concentration of 0.01 mM in the total 5 l volume. Following CBX administration, the pacing protocol was repeated with contact EGM recordings obtained at the same sites as baseline.

### Electrogram post-processing and analysis

We developed signal processing algorithms to automatically extract characteristics of the electrogram morphology for every activation in the dataset in a consistent manner. The algorithm was adapted for the intact heart from the one we previously developed for the analysis of myocardial cell monolayers [3].

A broad range of EGM signal time-domain features were extracted for each individual recording. The 19 features included were selected due to their known and established importance in cardiac electrophysiology from EGM and ECG interpretations, or as a region of interest previously investigated with myocardial cell monolayers that may provide further mechanistic insight [3]. To validate the accuracy of the automated interpretation, randomly selected EGMs were manually interpreted and compared with the feature extraction values.

### Statistical analyses

Statistical analyses were carried out using the Prism 5.0 software (GraphPad). Unless otherwise stated, *T* tests were performed to compare BL with CBX and *p* < 0.05 was considered statistically significant.

## Results

### Stability of the Langendorff model

All intact porcine and human hearts were successfully restarted (*n* = 13). The preparations remained stable and allowed all desired measurements and modulations to easily be performed in the time duration of each experiment (Table 2). Heart 6 had an experiment duration of 34 min because despite high preparation stability, it had to be terminated prematurely due to external factors. A total of 5492 individual beats were measured and analysed.

**Table 2:** The experiment duration for all whole heart Langendorff experiments performed (*n* = 13)

Heart	Duration of experiment (minutes)	Type of heart
1	89	Porcine
2	88	Porcine
3	117	Porcine
4	116	Porcine
5	116	Porcine
6*	34	Human donor
7	102	Porcine
8	90	Porcine
9	85	Human donor
10	88	Porcine
11	78	Human donor
12	98	Porcine
13	182	Human donor

### Capture of electrophysiological data

**Fig. 2:**
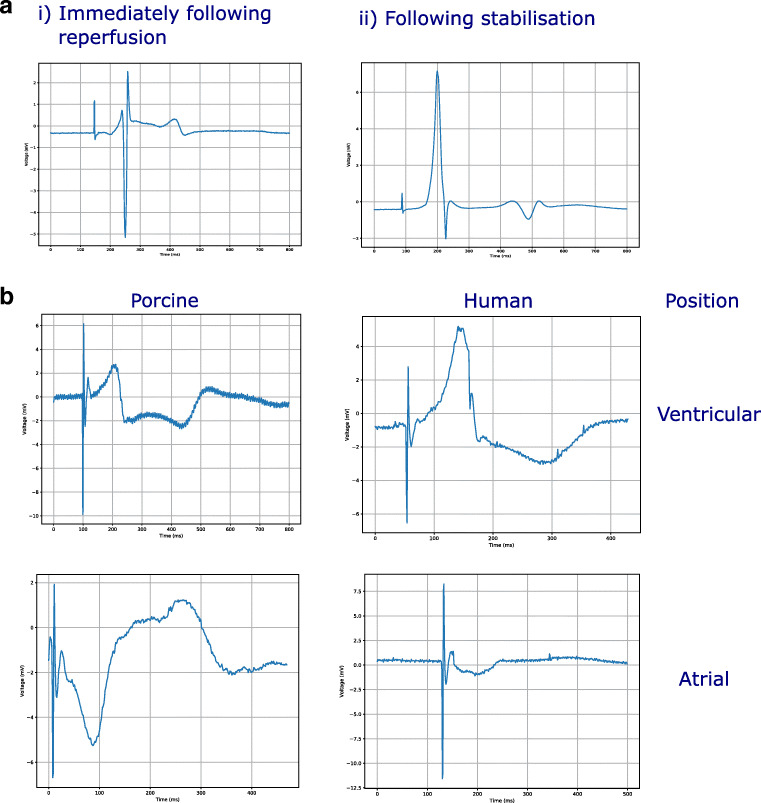
**a** Example ECG (i) when initially restarted. (ii) Reduction in ischemic features following stabilisation. **b** Extracted unipolar EGM traces from porcine and human hearts, taken at baseline. The porcine and human recordings were made from the same anatomical locations in the left ventricle and left atrium

When initially restarted, ST elevation was observed (Fig. 2a), with reduction in the ischemic features after 15 min of stabilisation (Fig. 2a). high-quality unipolar EGMs were successfully obtained from the epicardial surface of the left ventricle and left atrium (Fig. 2b) in both the porcine and human hearts.

### Pharmacological modulation of model

**Fig. 3:**
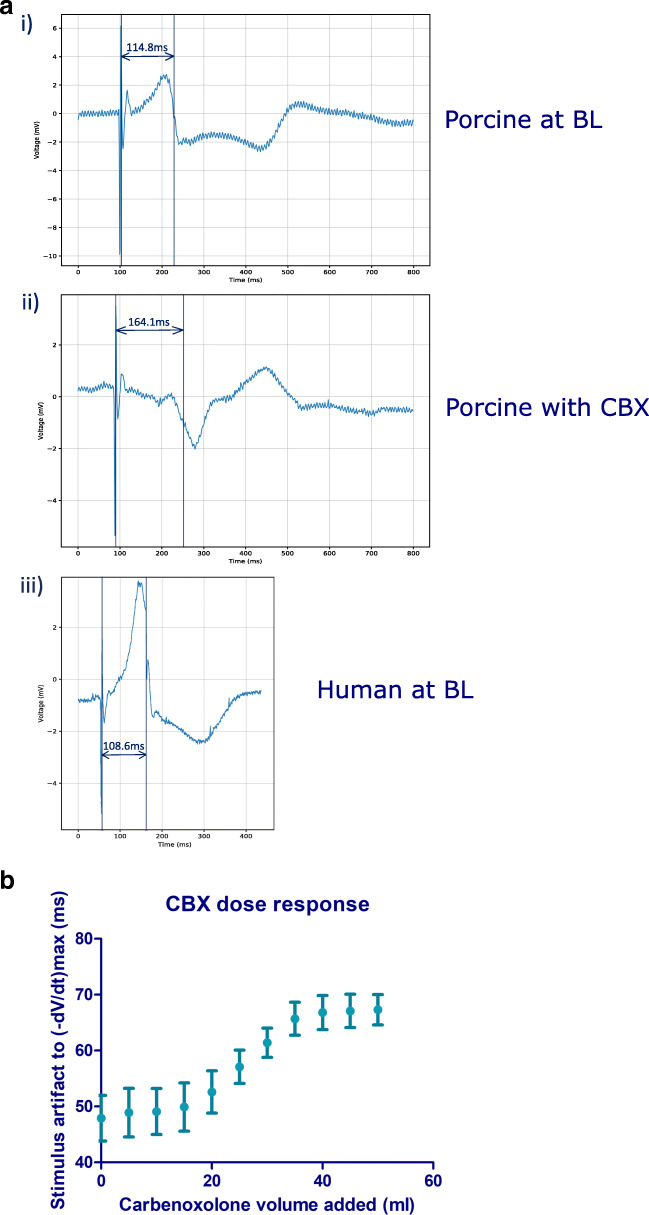
**a** Electrogram traces from a porcine and human donor heart, recorded from the same catheter position and paced at 750 cl. (i) The (-dV/dt)_max_ was measured 114.8 ms after the pacing stimulus, recorded with a porcine heart at BL. (ii) The (-dV/dt)_max_ was measured 164.1 ms after the pacing stimulus, recorded with a porcine heart after 50 ml of 1 mM CBX administered over 10 min. (iii) The (-dV/dt)_max_ was measured 108.6 ms after the pacing stimulus, recorded with a human donor heart at BL. **b** Dose response curve for CBX. The mean time delay and standard deviation from the pacing stimulus to the (-dV/dt)_max_, measured from all electrodes in contact with the myocardium. The progression of gap junction uncoupling caused by the administered CBX can be seen by the increased time delay from the pacing stimulus to the (-dV/dt)_max_ of the myocardium as more CBX is administered

Initial assessment of the response to CBX for the progression of cellular uncoupling on the EGM was by measuring the increase in the time interval between the pacing stimulus to time of (-dV/dt)_max_ following the administration of 50 ml of 1 mM CBX over 10 min (Fig. 3a). The EGM morphology and time interval between pacing stimulus to time of (-dV/dt)_max_ of the donor heart at BL are closer to the porcine heart at BL than following the administration of CBX (Fig. 3a). A mean interval of 47.9 ± 4.1 ms was observed across all electrodes at BL, whilst this interval increased to a mean of 67.2 ± 2.7 ms after 50 ml of 1 mM carbenoxolone was administered. The progression of CBX over the 10 min at 5 ml/min established the maximal response, with 50 ml dose (equivalent to 30.735 mg) being selected for all subsequent analyses, as this represented the maximal response, to investigate a pronounced feature change (Fig. 3b).

### Comparison of features between baseline and with carbenoxolone administered

**Fig. 4:**
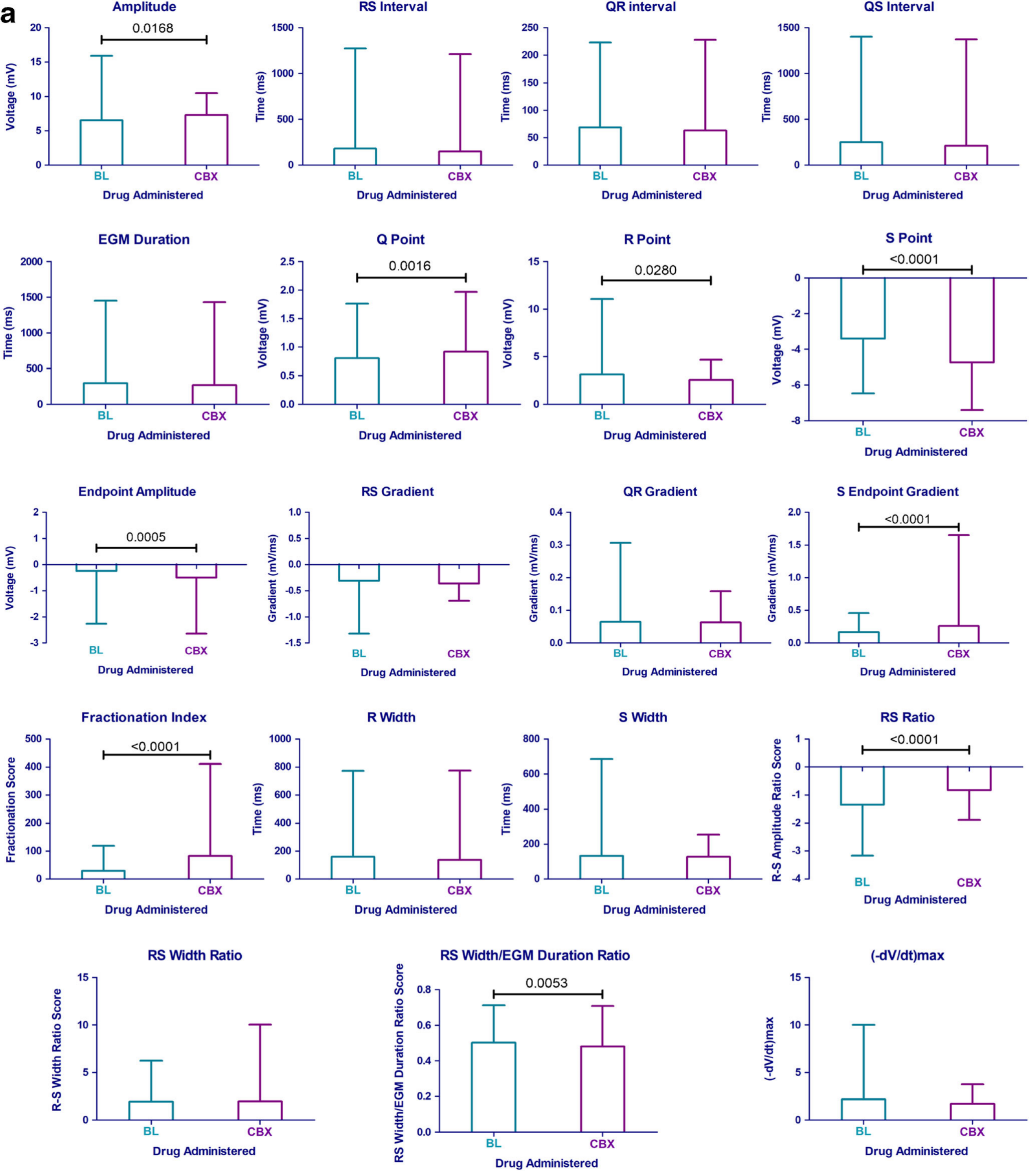

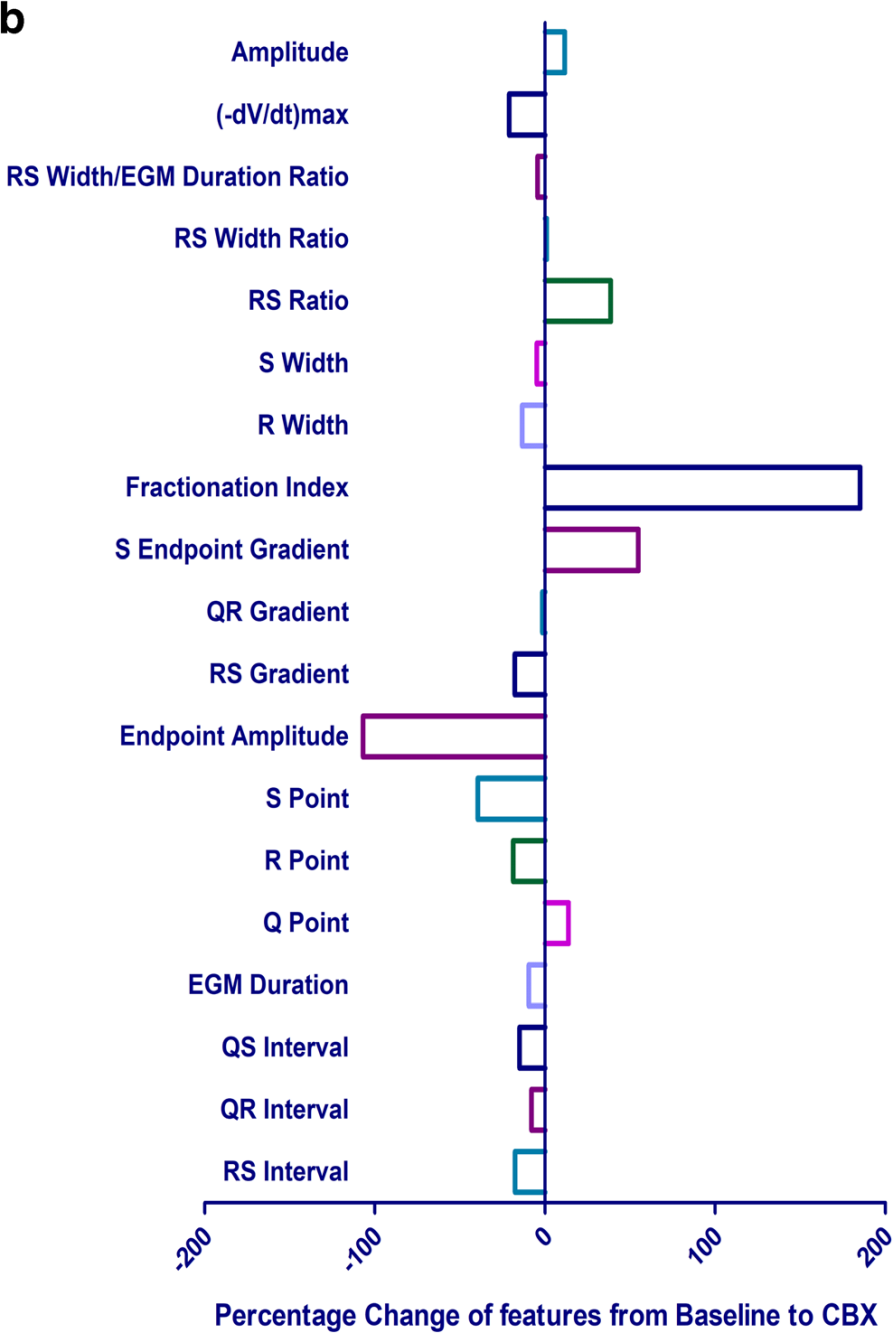
**a** The mean and SD of each feature extracted at BL and with CBX added. **b** The percentage change in EGM features from BL following the administration of 1 mM CBX

Following the automated extraction of the EGM characteristics at BL and with CBX added, the mean value and SD for each feature were calculated (Table 3). Q point amplitude, R point amplitude, S point amplitude, endpoint amplitude, S-endpoint gradient, fractionation index, RS ratio, RS width/EGM duration and amplitude were found to show statistical significance between BL and CBX (Fig. 4a).

**Table 3:** The mean, standard deviation and percentage change of each feature at BL and with CBX. *T* tests have been performed to determine if the changes in features are statistically significant

Feature	BL mean (SD)	CBX mean (SD)	*p* value	Percentage change (%)
RS interval	182.01 (1093.43)	149.96 (1061.53)	0.42	− 17.61
QR interval	68.59 (154.71)	63.21 (164.99)	0.34	− 7.84
QS interval	250.60 (1151.87)	213.17 (1159.46)	0.37	− 14.94
EGM duration	292.98 (1155.80)	265.20 (1163.59)	0.51	− 9.48
Q point	0.81 (0.96)	0.92(1.05)	0.002*	13.86
R point	3.13 (7.92)	2.55 (2.12)	0.03*	− 18.60
S point	− 3.40 (3.06)	− 4.74 (2.66)	*p* < 0.0001*	− 39.40
Endpoint amplitude	− 0.24 (2.02)	− 0.50 (2.14)	0.0005*	− 106.85
RS gradient	− 0.31 (1.01)	− 0.36 (0.33)	0.11	− 17.79
QR gradient	0.06 (0.24)	0.06 (0.10)	0.91	− 1.49
S-endpoint gradient	0.17 (0.29)	0.26 (1.40)	*p* < 0.0001*	54.93
Fractionation index	29.27 (89.76)	83.52 (327.44)	*p* < 0.0001*	185.30
R width	159.59 (613.57)	138.19 (635.67)	0.34	− 13.41
S width	133.39 (553.10	127.01 (127.01)	0.75	− 4.78
RS ratio	− 1.35 (1.81)	− 0.83 (1.06)	*p* < 0.0001*	38.58
RS width ratio	1.95 (4.28)	1.97 (8.06)	0.91	1.04
RS width/EGM duration	0.50 (0.21)	0.48 (0.23)	0.005*	− 4.30
(-dV/dt) max	2.17 (7.83)	1.72 (2.01)	0.08	− 20.94
Amplitude	6.53 (9.39)	7.28 (3.21)	0.02*	11.58

The features with the largest percentage change from BL to with CBX were fractionation + 185.3%, endpoint amplitude − 106.9%, S-endpoint gradient + 54.9%, S point − 39.4%, RS ratio + 38.6% and (-dV/dt)_max_ − 20.9% (Fig. 4b).

## Discussion

We have demonstrated the ability to successfully restart and sustain intact human and porcine hearts for over 3 h using our custom Langendorff preparation, showing its viability to be used as an ex vivo translational model to perform a variety of electrophysiological investigations. Contact EGM recordings were recorded from the hearts over this duration and pharmacological modulators can be used to investigate pathological substrates.

Until recently, the only human myocardium available for ex vivo analysis, if removed at the time of cardiac surgery, was very small samples of diseased human myocardium. Whilst providing essential information on cellular level activity, these samples have very little scope for comparison against a healthy myocardium and could not be used to investigate organ-level physiological processes and disease states that rely on the coordination, or mal-coordination, of different areas of the myocardium. Therefore, for a comprehensive analysis of these pathologies, intact preparations are necessary. There is clear evidence to suggest that large animal cardiac models more closely approximate human physiology than cell and animal models [6, 22] and there is a general acceptance that porcine hearts are similar to human [5, 19]. However, there are still some minor anatomical differences to consider, along with other factors such as immunological tolerance [10]. Despite these differences, we have demonstrated the development of an acute pathological model in the intact ex vivo human heart.

### Stability of preparation

We have demonstrated the use of the Langendorff apparatus for translational research with ex vivo human and porcine hearts. It enables the acquisition of unprecedented high-fidelity electrophysiological data in a manner that is otherwise impossible, giving rare insight into human disease states in a controlled ex vivo setting and allowing us to perform high resolution, controlled electro-anatomical characterisation. This system can maintain porcine and human whole hearts in a stable preparation for a duration sufficient to perform all planned data acquisition and can therefore be used to help provide a greater understanding of the electrophysiological structure and function of the heart. Although all the human donor hearts received were deemed unsuitable for transplant, there were variations in the cause of death, reason for rejection and consequently the condition of the hearts received (Table 1). Heart 11 was deemed unsuitable for transplant due to poor cardiac function, but the heart was successfully restarted and sustained on the Langendorff for an experiment length of 72 min, where all protocols were completed. The maximum investigation length recorded was 182 min on heart 13, at which point the human preparation was still stable and the duration was only limited by the time scale needed to complete the experimental protocol. This demonstrated that although the human hearts were unsuitable for transplants, they possessed the stability required for the study. The shortest recorded length was 34 min from heart 6. Despite the high stability of the preparation, it had to be terminated prematurely due to staff health and safety concerns. There was a possibility that a class 2 pathogen may have been present in the heart, which was not indicated until after initiation of the experiment. The stability of the preparation beyond the current experimental investigation protocol length allows for further study to expand beyond the scope of the existing protocol, with plans to include additional methodologies to the data acquisition.

### Utility of the preparations as an electrophysiological and contractility model

The ability to restart human and porcine hearts in a stable environment allows for finely controlled experimental conditions and the use of techniques to a higher fidelity than is possible in vivo*.* A stable controllable environment coupled with large intact hearts forms a closer translational model than is possible with cell or small animal models. The ex vivo model has the potential to be used for a variety of electrophysiological investigations, such as optical mapping for cellular electrophysiology, cardiac autonomic innervation and engineered heart tissue implantation to repair heart tissue damage. The preparation also enables investigations beyond electrophysiology, such as heart contractility and valve research to improve cardiac device design [8].

### Pharmacological modulation

We have previously used pharmacological modulators in simpler tissue and cell models to demonstrate modifications to ion channels and cellular coupling can be interpreted in the EGM [4]. Investigating these abnormalities in ex vivo intact hearts may provide insight into arrhythmogenic mechanisms. CBX causes cellular uncoupling and therefore artificially reproduces a pro-arrhythmic substrate. We delivered 50 ml of 1 mM CBX retrogradely into each heart via a bolus to the aorta using a syringe driver at a rate of 5 ml/min for 10 min. An alternate method of delivery for CBX would be to switch to a second 5-l reservoir filled with CBX-infused Tyrode’s solution at the final concentration. The bolus delivery method was preferred due to the direct delivery of the CBX to the aorta allowing for more precise measurement and incremental delivery of dosage delivered over the 10 min of infusion, enabling investigation of the dose response. Analysis of the EGM has revealed that during the 10 min of CBX infusion, there is an increase in the delay of the action potentials, with progression of the delay following an s-curve (Fig. 3b). The characteristic EGM morphology represents the variability in the electric field recorded at the electrodes, produced by the movement of ions across myocardial cell membranes, and the subsequent propagation of the action potential [18]. Since the contact EGM measures the electrical activity of the local myocardium, changes in cellular electrophysiology manifest in the electrogram morphology. The effects of cellular uncoupling on the myocytes are visible on the contact electrogram and our delivery method of CBX gives the expected effect at the dosage administered. The method of delivery would work for other drugs, and their effect on the cellular electrophysiology would be represented on the EGMs.

### Feature analysis

Analysis of the features of the EGMs was performed as a percentage change from BL. This enabled the human hearts to be compared together and with the porcine hearts. Performing the analysis in this manner meant that there was greater confidence that the changes we observed are a result of the CBX administered. When comparing the EGM of a donor heart at BL to a porcine heart at BL or with CBX (Fig. 3a), the EGM morphology of the donor heart was closer to the porcine heart at BL, suggesting that the donor hearts do not present with pro-arrhythmic substrates at baseline. The interval from the stimulus artefact to (-dV/dt)_max_ was 108.6 ms for a human donor heart at BL, 114.8 ms with a porcine heart at BL and 164.1 ms with a porcine heart after CBX was administered. The features of the EGMs with the largest percentage change found from BL to with CBX were the fractionation index, endpoint amplitude, S-endpoint gradient, S point, RS ratio and (-dV/dt)_max_. Fractionation is a feature of the electrogram which is measured clinically [20] and is thought to be associated with cell-to-cell discontinuity. Therefore, it is unsurprising that cellular uncoupling leads to an increase in fractionation, and is consistent with our previous findings [4].

We have identified further features of the electrogram not previously associated with cellular uncoupling. Due to their large change in morphology with cellular uncoupling, these features could be used when interpreting the EGMs to identify the underlying pathology. The underlying electrophysiological dysfunction associated with atrial fibrillation and other arrhythmias cannot be characterised solely by gap junction uncoupling; to establish the relationship between the changes in EGM morphology and arrhythmia, the other substrates associated with arrhythmia must also be investigated.

Our Langendorff apparatus can be used to administer other pharmacological modulators to investigate other arrhythmogenic substrates, such as ion channel blockers [4]. Identifying the features with most significant changes from BL in these EGMs could be used to establish collectively which combination of features differentiate between normal and different specific pathological EGMs, leading to a greater understanding of how pathological myocardial electrophysiological function manifests in the clinically accessible EGM. To leverage the analysis of the EGMs, there is potential to apply machine learning techniques to automatically distinguish more subtle changes in EGM morphology between a variety of underlying pathologies [3]. Due to the ex vivo preparation’s similarities to patients, with regard to size and baseline electrophysiological properties, the machine learning model could be applied to detect the same subtle changes in in vivo EGMs. This has the potential to be translated into a diagnostic tool by targeting regions of functional abnormalities during ablation procedures to treat arrhythmias by providing clinicians with real-time analysis of the EGMs they are collecting.

### Limitations

A possible limitation of this model is that the human donor hearts we receive have been deemed unsuitable for transplant, due to acute abnormalities, in contrast to the healthy porcine hearts. These donor hearts therefore may have a different baseline to healthy human hearts. We have attempted to correct for this by using a percentage change from BL, to account for any intrinsic BL differences. Clinically, EGMs are routinely recorded from the endocardial surface. However, in the absence of clinical 3D electroanatomic mapping systems, precise endocardial positioning of the catheter would be challenging on the heart ex vivo. We have instead recorded from the epicardial surface for better control over catheter positioning and so that the positioning can also be easily recorded with photographs for reference.

## Conclusion

In this study of ex vivo intact human and porcine hearts, we have demonstrated that we can restart, maintain, pharmacologically modulate and electrophysiologically interrogate these large hearts using a custom-built Langendorff apparatus, along with the possibility to expand the scope of existing experiments further, such as the inclusion of optical mapping. The effect of cellular uncoupling is visible on the EGM. With the administration of other pharmacological modulations to reproduce other pro-arrhythmic substrates by ion channel blockade, the features of the EGM have the potential to differentiate between normal and pro-arrhythmic and could be of benefit to guide ablation procedures.

## Data Availability

All data supporting the findings of this study are available within the article and from the corresponding author on reasonable request.
